# System Dynamics Models of Online Lending Platform Based on Vensim Simulation Technology and Analysis of Interest Rate Evolution Trend

**DOI:** 10.1155/2022/9776138

**Published:** 2022-08-09

**Authors:** Sulin Pang, Chao Deng, Susong Chen

**Affiliations:** ^1^Institute of Finance Engineering at School of Management, School of Emergency Management, Jinan University, Guangzhou 510632, China; ^2^School of Emergency Industry, Guangdong Innovative Technical College, Dongguan, Guangdong 523960, China; ^3^Guangdong Emergency Technology Research Center of Risk Evaluation and Prewarning on Public Network Security, Guangzhou 510632, China; ^4^Guangdong Rural Credit Union, Guangzhou 510630, China

## Abstract

This paper mainly focuses on the construction of a system dynamics model of an online lending platform and the application of Vensim simulation technology in the analysis of the evolution trend of the investment interest rate. The system dynamics flow graph models of the investment subsystem, loan subsystem, and interest rate subsystem of the online lending platform were innovatively constructed; the level variable, flow rate variable, auxiliary variable, and mathematical relations between these variables as they relate to the platform were innovatively studied. Based on the key variables of the online lending platform, a system dynamics model of the online lending platform was innovatively constructed. The parameters were assigned using data from China's online lending industry, and the logical consistency, sensitivity, and validity of the simulation data of the system dynamics model were tested. Finally, we used Vensim simulation technology to simulate and analyze the impact of emergency scenarios on the interest rate evolution trend. Overall, this paper provides a scientific simulation technology and data analysis method for examining online lending platforms.

## 1. Introduction

Online lending platforms are a phenomenon in great expansion around the word in the last decade. This paper summarizes the current research results and finds that there is a lack of research on the evolution mechanism of the interest rate of online lending platforms. In order to find the interest rate evolution mechanism of online lending platforms, we use Vensim simulation technology to innovatively construct a system dynamics model of online lending platforms, and verify the model. The results show that the model has practical significance. This paper applies the model to simulate the interest rate change under the emergency scenario and innovatively finds the important law of interest rate cyclical change.

In March 2005, the first online lending platform, named ZOPA (Zone of Possible Agreement), was developed in the UK [1]. Prosper, which was the first online lending platform in the US, was created in 2006 [2]. LendingClub, which was the second online lending platform in the US, was developed in 2007 [3]. Smava, which was the first online lending platform in Germany, was created in 2007 [4]. Online lending platforms have increasingly become an important part of human life since the development of these early platforms. The online lending industry has always been a focus of academics and the government. At present, the research on online lending platforms mainly focuses on the following aspects.

The first aspect consists of the characteristics of the online lending industry. Mariotto [3] believed that an online lending platform is a bilateral market in which investors and borrowers interact with each other. The more investors there are, the more abundant funds there will be, and the more borrowers will be attracted. At the same time, the more borrowers there are, the greater the demand for capital will attract more investors. Ashta and Assadi [4] conducted an empirical study on the development of the online lending industry in Europe and analyzed the situation of online lending platforms in the UK, Netherlands, Denmark, Italy, Poland, France, and other countries. The main service objects of online lending platforms in Europe include small and microenterprises and people who cannot obtain financial services from traditional financial institutions. Loan rates range from 1% to 22%, lender fees range from 0.5% to 1.5% of the transaction amount, and service fees range from 0.5% to 4%. Dhand et al. [5] defined online lending as Internet-based social lending in which the service objects are high-risk groups excluded by traditional banking institutions, such as those who are not satisfied with the interest rate of traditional financial storage or who have low credit ratings and no stable income. The data analysis results of ZOPA show that social lending fills the gap between microfinance and traditional lending. Bachmann et al. [6] analyzed the stakeholders of online lending platforms, including internal managers, general employees, and shareholders of the platform and external regulators, investors, borrowers, community organizations, bank cooperative institutions, credit rating agencies, and small loan institutions. Yu and Shenwei [7] argued that online lending belongs to the sharing economy, which improves the utilization rate of idle funds and small funds and endows both lenders and borrowers with more free choices. Ding et al. [8] believed that China's online lending industry has the characteristics of a crowdfunding economy. Herrero-Lopez [9] believed that online lending is the application of microfinance in Internet communities and that online lending platforms provide interaction and meeting opportunities for lenders and borrowers.

The second aspect is the operation and management of online lending platforms. Mariotto et al. [3] studied the competition strategies of Prosper and LendingClub, which are two major online lending platforms in the United States, and performed comparative analysis from the aspects of loan amount, lender credit score, platform rate, loan interest rate, settlement method, and relationship with traditional banks. Mariotto et al. [3] believed that LendingClub adopted stricter conditions than Prosper in terms of lender screening, but there were great similarities in other aspects. Meanwhile, the online lending platform format did not have a great impact on the traditional banking industry but rather acted as a supplement to traditional banks. Suryono et al. [10] used the systematic literature review (SLR) method to sort out the problems or risks existing in online lending platforms from the aspects of registration, repayment, asset evaluation, investment decision, platform registration, and supervision and provided relevant solutions. Feller et al. [11] used social identity theory to analyze collective identity and social identity among online borrowers; studied the relationship between social identity, personal transparency, information sharing, and user behavior and the information sharing mechanism of online lending platforms; and conducted an empirical study on online lending platform information, which included credit rating, household income, loan history, bank loans, platform charges, and other information. Kaminskyi and Petrovskyi [12] carried out a simulation analysis of investors' capital flow, borrowers' behavior, credit risk, interest rate, and other factors in online small-sum consumer loans by the method of system dynamics and put forward suggestions for the daily operation of online small-sum consumer loans.

The third aspect is the success factor of online loan transactions. Bachmann et al. [6] studied the interest rate, credit rating, and borrower characteristics and the relationships between them. Prosper classifies borrowers according to their credit scores (520 to 900), including HR, E, D, C, B, A, and AA, which are ranked from low to high. The corresponding borrowing rate ranges from 25% to 7.5%. The higher the credit score is, the higher the credit rating is, and the lower the borrowing rate is. At the same time, empirical research on nationality, age (35–60), gender, and social network information (friends, pictures, associations, etc.) of the borrowers shows that the borrowing rate of single women is 0.4% lower than that of single men and the return on investment of single women is approximately 2% lower than that of single men. Herrero-Lopez [9] analyzed the lending data of the Prosper platform by the clustering method and studied the relationship between social interaction in social groups and online lending risks, especially one-to-one or one-to-many relationships. The research results showed that when loans could not be obtained, one can improve one's borrowing success rate by enhancing one's social features. Klafft [13] conducted an empirical analysis of 54,077 borrowers on Prosper, and the results showed that the authenticity of bank account information and credit rating were key factors for the success of loans, while personal information (such as photos of borrowers) also had an important impact on financing. Community information is correlated with loan outcomes, but the correlation is not significant. Klafft [13] believed that the interest rate of online lending platform is mainly determined by the credit rating and debt-to-income ratio and that borrowers with high credit ratings are more likely to obtain loans. Zhang et al. [14] conducted an empirical analysis using a dataset of PPDAI data, which was the first online lending platform in China; the authors studied the factors that determine the probability of obtaining loans in online lending. The research results showed that the annual interest rate, credit rating, number of successful loans, gender, and credit score of loans had a positive impact on loan success. In addition, repayment term, description, and number of failed loans had a negative impact on loan success; finally, it was found that men were more likely to obtain loans. Liu et al. [15] proposed a P2P lending recommendation (P2PLR) system, which pairs borrowers and investors according to their historical interaction information and identifies investors' preferences for risk and return awareness by establishing risk and return awareness models to improve lending efficiency. Chen et al. [16] analyzed the data of some online lending platforms in China from June 2017 to 2018 and believed that the interest rate of online lending platforms in China was not fully marketized, which was mainly determined by the platforms themselves and was also related to the loan term, number of new investors, repayment amount, and platform credit rating. He et al.[17] took 224 large online lending platforms in China from 2015 to 2019 as the research object to study whether investor attention will affect returns; the authors also used the Baidu search index as the quantitative index of investor attention. The empirical results showed that investor attention will reduce the average project interest rate of the platform.

The fourth aspect is supervision of the online lending industry. Yu et al. [7] studied the regulatory policies of the online lending industry in the United States and China and argued that the regulatory authorities should play the role of gatekeepers by focusing on verifying the authenticity, accuracy, integrity, and timeliness of information disclosure, while leaving enough space for financial innovation and avoiding excessive interference in platform operation; the authors developed a supervision system that is friendly, sensitive, and efficient for the utilization of idle capital. Ding et al. [8] studied the development of China's online lending industry from 2007 to 2019, as well as the online lending regulatory policies in China, the United States, and the United Kingdom. Ding et al. [8] argued that the financing needs of small- and medium-sized enterprises and residents' pursuit of high returns on capital have led to the rapid development of China's online lending industry and that financial innovation reduces transaction costs through automated means. To improve transaction efficiency, the government needs to encourage financial innovation, while special attention should be given to investor protection and social security and stability in financial innovation activities. Improper supervision will lead to a waste of economic resources.

The fifth aspect is lending credit risk. Suryono et al. [10] conducted an empirical study on the development of the online lending industry in Indonesia and found that there were risks related to data leakage and data access restrictions, including personal data protection, personal data fraud, illegal fintech lending, and product marketing ethics. Chen et al. [16] analyzed the data of some online lending platforms in China from June 2017 to 2018 and found that the higher the credit rating of the platform was, the lower the lending rate was; the authors also believed that the lending rate of an online lending platform could reflect the risks of the platform. Santoso et al. [18] studied the online lending industry in Indonesia and analyzed the lending data of the three major online lending platforms from 2014 to 2018. The authors [18] studied the lender characteristics (loan amount, loan term, marital status, income, age, gender, education degree, etc.), household characteristics, platform characteristics, and relationship between the interest rate and the condition of borrower default; they found that the analysis results of the different platforms are different, although interest rates are likely to be determined by the supply relationship between borrowing and lending, and supply will encourage mortgage rates to rise. Emekter et al. [19] evaluated the relationship between credit risk and loan performance based on data from LendingClub and found that credit rating, debt-to-income ratio, and FICO score were significantly related to the loan default rate. The longer the loan period was and the lower the credit rating of the borrower was, the greater the risk of loan loss was. Zhao et al. [20] used complex network theory and infectious disease dynamics theory to study the spread of credit risk on online lending platforms; the authors divided online lending platforms into four types, namely, susceptible platforms, risk platforms, infection platforms, and immune platforms, and they constructed an evaluation model of credit risk contagion on online lending platforms.

The last aspect consists of others. Najaf et al. [21] studied the impact of COVID-19 on the online lending industry in the United States. The results show that from January 2019 to June 2020, the amount of online lending transactions in the United States was significantly higher than the amount before the pandemic,with the average amount of loans being 23% higher. In addition, it was found that the average interest rate of online lending issued during the pandemic increased by 7% compared to that during the prepandemic period, while the average loan term also increased significantly.

In summary, the current research focuses on the characteristics of the online lending industry, online credit platform, network operation management, supervision, online credit risk, etc., and the related research theories and methods include the social identity theory, literature review method, complex network theory, dynamics theory of infectious diseases, shared economic theory, system dynamics theory, and empirical analysis. The existing research mainly focuses on the empirical analysis of the online lending industry; however, the research content largely ignores the operation mechanism and interest rate formation mechanism of online lending platforms and the dynamic analysis of the operation of online lending platforms. An online lending platform is a complex social financial technology system, and its operation is affected by the investors, borrowers, and policy environment and is a complex time-varying system. System dynamics theory is widely used to solve complex time-varying system problems. Therefore, system theory and system dynamics theory are selected as the main research tools used in this paper.

The main contributions of this paper are as follows. (1) The paper studies the system dynamics flow diagram model of online lending platforms, including investment subsystem, loan subsystem, and interest subsystem. (2) This article innovatively studies the three types of variables found on an online lending platform, namely, auxiliary, rate, and level, and the mathematical relationship between them. (3) The paper verifies the validity of the model by using data from China's online lending industry. (4) Finally, this paper conducts simulation analysis of the interest rate evolution trend of online lending platform.

The outline of rest of this article is as follows. In Section 2, the online lending platform system structure is established, including seven subsystems, namely, investment management, loan management, transfer of creditor rights, funding management, parameter management, lending process management, and backstage management. The system dynamic flow graph models of the online lending platform are studied, including the investment subsystem, loan subsystem, and interest rate subsystem.  Section 3 studies and defines three kinds of variables of the online lending platform, namely, auxiliary variables, rate variables, and level variables, as well as the mathematical relationship between them, and constructs a system dynamics model of the online lending platform.  Section 4 describes the algorithm design and implementation steps based on Vensim.  Section 5 verifies the correctness of the system dynamics model, including the logic consistency, sensitivity, and validity of the simulation data of the model, through one of China's online lending platforms. In Section 6, Vensim simulation technology is used to simulate the impact of emergency scenarios on the interest rate evolution trend and analyze the relevant data.  Section 7 presents the conclusion.

## 2. The System Analysis of an Online Lending Platform

### 2.1. The System Structure of an Online Lending Platform

According to system theory, an online lending platform is essentially a financial system that includes seven subsystems: investment management, loan management, transfer of creditor rights, funding management, parameter management, lending process management, and backstage management. The details are as follows:

1. The investment management subsystem is a system for investors to carry out account registration, real-name authentication, binding of platform accounts with bank accounts, screening of loan objects and investment objects, recovery of funding including principal and interest, and other business operations.
2. The loan management subsystem is a system for borrowers to carry out account registration, real-name authentication, binding of platform accounts with bank accounts, issuing of loan objects, repayment, and other business operations.
3. The subsystem of creditor's rights transfer is a system in which investors can recover the invested funds in advance. Its functions include applying for the transfer of creditor's rights, setting the price of creditor's rights, transferring creditor's rights, recovering investments, and purchasing creditor's rights transferred by other investors.
4. The fund management subsystem is a system used to manage the funds of investors and borrowers. Its functions include fund transfers and docking of management platform accounts with bank accounts, third-party payment accounts, and other external fund transfer channels.
5. The backstage management subsystem is mainly composed of the investment process management subsystem, the loan process subsystem, the member management subsystem, and the parameter management subsystem. Among them, the investment process management system is a subsystem for backstage managers to optimize the investment process.
6. The loan process subsystem is mainly used for backstage managers to optimize the loan process. The member management subsystem is used for backstage managers to carry out real-name authentication, qualification examination, pre-loan investigation, risk control audit, legal audit, and post-loan management.
7. The parameter management subsystem is used for backstage managers to adjust the loan interest rate, investment interest rate, loan term, loan amount, regulatory rules, and so on. It is used to maintain the stable operation of an online lending platform.

**Figure 1:**
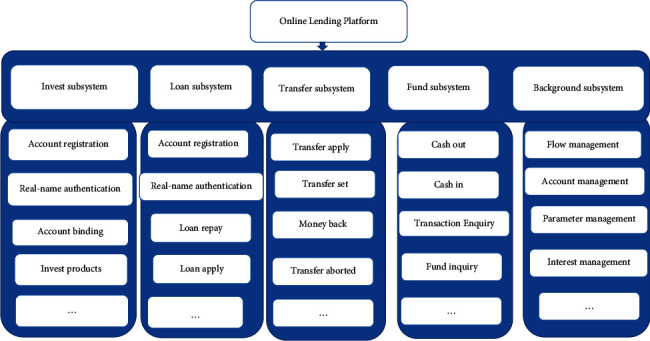
The system structure diagram of an online lending platform.

By analyzing the structure, function, and capital flow of an online lending platform, it can be found that some factors have an important impact on the operation of an online lending platform, including investors, borrowers, interest rates, loan amount, investment amount, and loan term (Figure 1).

### 2.2. System Dynamic Flow Diagram of an Online Lending Platform

#### 2.2.1. Investment Subsystem

**Figure 2:**
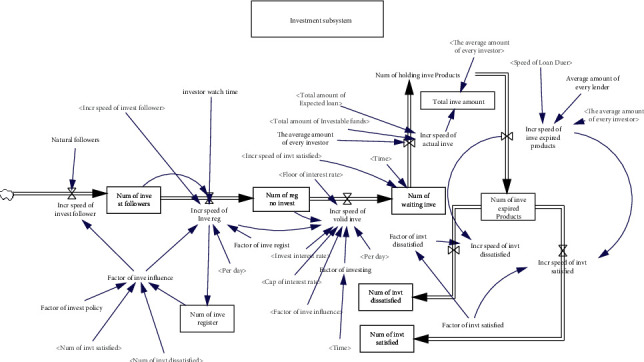
The dynamic flow diagram of the investment subsystem.

Compared with traditional banks, online lending platforms are new and risky. It usually takes a long time for investors to become familiar with the platform and invest their money, ranging from a few weeks to a few months. Investors generally obtain information from online lending platforms through the Internet, advertising, TV, acquaintances, and other channels and then learn about the relevant conditions of the platform, including investment risks, investment returns, and related products; thus, they become observers of online lending platforms. After understanding and observing, some observers will establish trust relationships with, submit registration information to, register on, and have a deeper understanding of the platform. Some registrants transfer funds to the platform for investment after studying and becoming familiar with the platform for a certain period of time. Some investors, whose funds are successfully recovered and whose satisfactory returns are obtained after the expiration of the investment project, have more faith in the online lending platform. They then give back to the platform by increasing the amount of investment or recommending others to invest on the platform. The platform and investors win and form a positive interaction. Some investors, whose income expectations cannot be met or whose principal is lost via investment projects in bad debts, withdraw from the platform and relate their poor investment experience to other investors through the Internet, the BBS, QQ, WeChat, and other forms of communication. The reputation of the platform is thus negatively affected. If too many of these things happen, this will lead to a vicious cycle. The dynamic flow diagram model of the investment subsystem is shown in Figure 2.

#### 2.2.2. Loan Subsystem

**Figure 3:**
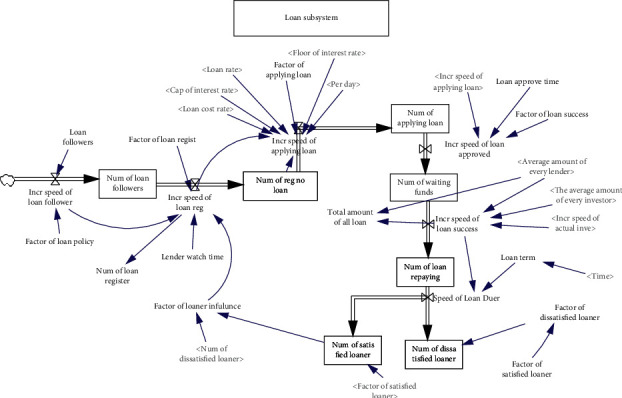
The dynamic flow diagram of the loan subsystem.

There are many similarities between the behaviors of lenders and investors on online lending platforms. The difference lies in the stronger purpose of lenders and the shorter period between knowing the platform and borrowing. All of these individuals obtain information on online lending platforms through the Internet, advertising, TV, acquaintances, and other means. Their purposes complement each other, with investors lending money for profit and lenders paying interest for access to funds for a fixed period of time. After obtaining a certain understanding of the loan conditions and relevant requirements of the online lending platform, the borrower can register on the platform to gain a deeper understanding of the platform. Some registrants will prepare relevant loan materials and apply for loans after studying and becoming familiar with the platform for a certain period of time. After the project expires, some of the borrowers will smoothly return the funds, have a quick and convenient loan experience with the platform, and thereby form a positive interaction with the platform. However, after the loan project expires, some borrowers cannot return the funds on schedule or are required to repay the funds in advance and thus do not have a good investment experience. The dynamic flow diagram model of the loan subsystem is shown in Figure 3.

#### 2.2.3. Interest Rate Subsystem

**Figure 4:**
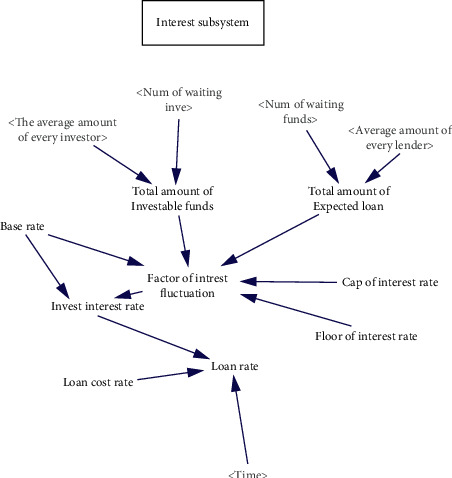
The dynamic flow diagram of the interest rate subsystem.

The main function of the online lending platform is to match the supply and demand of currency, while the interest rate, which is determined by the supply and demand of currency, reflects the cost of capital. The supply and demand of currency is mainly affected by the number of investors, the amount of investable funds, the number of borrowers, the expected financing amount, the platform cost rate, the platform interest rate, the management strategy, and so on. Investors and borrowers have opposite expectations for interest rates. Investors expect to obtain high returns through high interest rates, while borrowers expect to pay fewer capital costs to obtain the right to use capital. Online lending platforms regulate interest rates by setting the highest interest rate, the lowest interest rate, and the benchmark interest rate so that investors and borrowers can reach a consensus. The dynamic effect of the interest rate subsystem is shown in Figure 4.

## 3. The System Dynamics Model of an Online Lending Platform

### 3.1. Variable Hypothesis

To establish the system dynamics model of the online lending platform, three types of variables were assumed in the Vensim simulation system, namely, auxiliary variables, rate variables, and level variables. Their symbols and meanings are as follows.

#### 3.1.1. Auxiliary Variables

In this paper, auxiliary variables are used to represent the conversion ratio parameters of all kinds of investors and borrowers, as well as the time parameters of investment decisions, loan approvals, policy regulations, and other influencing parameters. The symbols of auxiliary variables and their meanings are as follows.

Suppose *In*_*lka* represents normal concern numbers, which refers to the number of potential investors who pay attention to the platform in general; these people are likely to register as investors. *In*_*pal* represents the investment policy regulation coefficient, which refers to the impact of policies on investors. A positive impact will lead to an increase in the rate of investment wait-and-see, while a negative impact will lead to a decrease in the rate of investment wait-and-see. *In*_*lkt* represents investment wait-and-see time, which refers to the time when a wait-and-see investor converts to a registered one. *In*_*rgk* represents investor registration coefficient, which refers to the proportion of investors who are on the sidelines of investment and are converted into registrants under normal circumstances. *In*_*wtk* represents the investment coefficient, which refers to the proportion of registered non-investors who are converted into actual investors. *In*_*sak* represents investor satisfaction coefficient, which refers to the proportion of investors who are satisfied with the service of online lending platform. *In*_*dsk* represents investor dissatisfaction coefficient, which refers to the proportion of investors who are not satisfied with the service of online lending platform. *Ae*_*in* represents the per capita investment amount, which refers to the average investment amount of each investor on the online lending platform. *Ln*_*lka* represents the number of loan watchers per day, which refers to the number of new loan watchers per day under normal circumstances. *Ln*_*lkt* represents loan wait-and-see time, which refers to the interval between wait-and-see and registration on the platform. *Ln*_*rgk* represents the borrower registration coefficient, which refers to the proportion of people registered on the platform among loan watchers. *Ln*_*apk* represents the loan application coefficient, which is the percentage of registered borrowers who apply for loans. *Ln*_*tm* represents the term of the loan, which is how long the borrower borrows money. *Ln*_*ir* represents the loan interest rate, which refers to the actual interest rate of online lending borrowers. *Ln*_*ifk* represents the borrower influence coefficient, which refers to the degree of influence that those who have successfully taken out loans have on those who are waiting to take out loans. *Ln*_*avm* represents per capita loan amount, which refers to the average loan amount of each borrower on the online lending platform. *Ln*_*epa* represents the total amount of expected financing, which refers to the total amount of loans expected by all borrowers on the online lending platform. *Ln*_*a*  *dk* represents the loan approval factor, which is the percentage of loan applicants who are approved. *Ln*_*a*  *dt* represents the loan approval time, which refers to the interval between the loan application and the loan approval. *Ln*_*sak* represents the satisfied borrower coefficient, which refers to the proportion of people who have borrowed money from the platform and are satisfied with the platform. *Ln*_*ds*  *k* represents the unsatisfied lender coefficient, which refers to the proportion of people who have borrowed money from the platform and are not satisfied with the platform. *Ln*_*plk* represents the policy coefficient of online lending, which refers to the influence of policies on online lending. If the value of this variable is greater than 1, this represents a positive impact, which will lead to an increase in online lending trading volume; if it is less than 1, this represents a negative impact, which will lead to a decline in online lending trading volume. *Ir*_up represents the highest interest rate, which refers to the upper limit of the interest rate set by an online lending platform. *Ir*_*dn* represents the lowest interest rate, which refers to the lower limit of the interest rate set by an online lending platform. *Ir*_*ref* represents the benchmark interest rate, which refers to the reference interest rate set by the online lending platform. *Ir*_in represents the investment rate, which is the real interest rate of the investor. *Ir*_*flu* represents the rate volatility coefficient, which describes the amount of difference between the actual investment rate and the benchmark rate. In_*fuk* represents investor influence coefficient, which is used to describe the influence of the platform's existing registered investors on potential investors. *Pf*_*rt* represents the platform rate, which refers to the annualized service rate charged by the online lending platform for each loan. In_*asvh* represents the total investable amount, which refers to the total amount of funds that can be lent on the online lending platform.

#### 3.1.2. Flow Rate Variables

In this paper, flow rate variables are used to represent the change rate of the number of different kinds of investors and borrowers, as well as the approval rate in the loan process. Their symbols and meanings are as follows.

Suppose *In*_*lkr* represents the increase rate of wait-and-see investments, which refers to the increasing number of daily wait-and-see investments on the platform. *In*_*rgr* represents the increase rate of daily investment registrants, which refers to the increasing number of registered investors on the platform. *In*_*wtr* represents the increase rate of daily effective investors, which refers to the increasing number of waiting investors on the platform. *In*_*hdr* represents the increase rate of daily actual investors, which refers to the number of actual investors who lend out funds. *In*_*sar* represents the increase rate of daily satisfied investors, which refers to the increasing number of investors who are satisfied with the platform after the project expires. *In*_*dsr* represents the increase rate of daily unsatisfied investors, which refers to the number of investors who are unsatisfied with the platform after the expiration of the project. *In*_*eps* represents the increase rate of daily investment project maturity, which refers to the increasing number of investors with maturing projects. *In*_*lkr* represents the increase rate of wait-and-see loans, which refers to the increasing number of daily wait-and-see loans that are found on the platform. *In*_*fuk* represents the growth rate of daily loan registrants, which refers to the number of new borrowers on the platform. *In*_*apr* represents the rate of loan applicants, which is the daily number of borrowers applying for loans. *In*_*wtr* represents the rate of loan approval, which refers to the daily number of borrowers approved for loans. *Ln*_*rpr* represents the rate of increase of successful borrowers, which is the daily number of borrowers who successfully receive loans. *Ln*_*exr* represents the speed of loan maturity, which refers to the daily number of borrowers who have loan maturity on the platform.

#### 3.1.3. Level Variables

In this paper, level variables are used to represent the cumulative number of different kinds of investors and borrowers and the capital amount of loans. Their symbols and meanings are as follows.

*In*_*lk* represents the cumulative number of investors who are interested in P2P online lending investment but have not registered on the platform. *In*_*rg* represents the cumulative number of registered non-investors. *In*_*wt* represents the cumulative number of waiting investors who plan to invest but have not yet invested; once an investment product is released, then such investors will invest. *In*_*hd* represents the cumulative number of holders of investment products who are waiting for the expiration of the invested projects. *In*_*sa* represents the cumulative number of satisfied investors who have invested in the platform and are satisfied with the platform, while *In*_*ds* represents the cumulative number of unsatisfied investors who have invested in the platform and are not satisfied with the platform. *In*_*am* represents the cumulative investment amount, which refers to the total amount of funds invested by investors in the platform. *Ln*_*lk* represents the cumulative number of loan wait-and-see borrowers who are potential borrowers. *In*_*ar* represents the total number of investment registrants. *Ln*_*rg* represents the total number of borrowers who have registered for loans. *In*_*rp* represents the total number of borrowers who have received a loan but have not repaid that loan. *In*_*ep* represents the total number of borrowers with project maturity. *In*_*ap* represents the total number of borrowers applying for loans. *Ln*_*sa* represents the total number of borrowers who have borrowed money and are satisfied with the platform, while *Ln*_*ds* represents the total number of borrowers who have borrowed money and are not satisfied with the platform. *Ln*_*ar* represents the cumulative number of registered borrowers. *Ln*_*am* represents the cumulative loan amount. *Ln*_*wt* represents the total number of borrowers who have been approved for a loan and are waiting to receive funding.

#### 3.1.4. Variable Summary Table

Now, we have completed the definition of the three types of variables, as detailed in Table 1.

### 3.2. The Mathematical Formula between Variables

In this paper, Vensim modeling software was used for system dynamics modeling. The variables commonly used in Vensim mainly included three types: auxiliary variables, rate variables, and level variables. The mathematical relationship between variables is expressed as follows:

(1) $$\begin{array}{c} \text{Level}\left(t\right)=\int_{0}^{t}\left(\text{Rate}\right)+\text{Auxilary}. \end{array}$$

Formula (1) shows that the level variable is the integral of the flow rate variable, which reflects the result of state change; its initial value can be expressed by an auxiliary variable.

(2) $$\begin{array}{c} \text{Rate}\left(t\right)=\frac{d\left(\text{Level}\left(t\right)\right)}{d\left(t\right)}. \end{array}$$

Formula (2) shows that the flow rate variable is the differential of the level variable with respect to time, which reflects the rate of state change.

(3) $$\begin{array}{c} \text{Rate}\left(t\right)=F\left({\text{Auxilary}}_{0,\ldots ,}{\text{Auxilary}}_{j}\right). \end{array}$$

Formula (3) shows that flow rate variables can be calculated by auxiliary variables.

(4) $$\begin{array}{c} {\text{Auxilary}}_{i}=F\left({\text{Auxilary}}_{0,\ldots ,}{\text{Auxilary}}_{j}\right)|\left(i\neq j\right). \end{array}$$

Formula (4) shows that there is a functional calculation relationship between auxiliary variables.

### 3.3. The Construction of the System Dynamics Model

Based on the results of the system analysis of the online lending platform, this section constructs the system dynamics model of the online lending platform. Vensim modeling software was used to define the formula between variables. The main functions used include INTEG(), XIDZ(), DELAYMATERIAL(), MAX(), MIN(), and INTEGER(). INTEG() represents the integration of the flow rate variables, that is, the accumulation of flow rate variables. XIDZ() represents the division relationship of the variables, and we can specify the return value when the denominator argument is 0. DELAY MATERIAL() represents the delay function, which is used to represent the delayed transmission of information. For example, an investor on the sidelines can register after 5 days, which can be described through this function. MAX() represents the maximum value of the function argument. MIN() represents the minimum value of the function argument. INTEGER() is used to round the variables.

(1) The system dynamics model of auxiliary variable formulas is as follows:
  (5) $$\begin{array}{c} \text{In\_avsh}=\text{In\_}wt\times Ae\text{\_in}. \end{array}$$
Formula (5) shows that the total investable amount is the product of the number of waiting investors and the average investment amount.
  (6) $$\begin{array}{c} \text{In\_}fuk=\text{XIDZ}\left(\text{In\_}sa\text{,In\_}ar\text{,}0\right)-XI\;DZ\left(\text{In\_}ds\text{,In\_}ar\text{,}o\right)+1+\text{In\_}pla. \end{array}$$
Formula (6) shows that the investor influence coefficient is the sum of the difference between the proportion of satisfied investors and the proportion of dissatisfied investors, the policy regulation coefficient, and 1. A value of 1 means that policy and investor satisfaction have no influence on investors. To simplify the study, when the policy regulation coefficient is 0 and the proportion of satisfied investors is larger than that of dissatisfied investors, the investor influence coefficient is larger than 1, which indicates a positive influence.
  (7) $$\begin{array}{c} \text{In\_}ds\;k=1-\text{In\_}sak. \end{array}$$
Formula (7) shows that the investor dissatisfaction coefficient is 1 minus the investor satisfaction coefficient.
  (8) $$\begin{array}{c} Ln\_ds\;k=1-Ln\_sak. \end{array}$$
Formula (8) shows that the dissatisfied lender coefficient is 1 minus the satisfied lender coefficient.
  (9) $$\begin{array}{c} Ln\_epa=Ln\_wt\times Ln\_\text{avm}. \end{array}$$
Formula (9) shows that the total amount of expected loans is the product of the number of borrowers waiting for funds and the average amount of loans.
  (10) $$\begin{array}{c} Ln\_ir=Pf\_rt+Ir\_\text{in}. \end{array}$$
Formula (10) shows that the loan interest rate is the sum of the platform cost rate and investment interest rate.
  (11) $$\begin{array}{c} Ln\_ifk=\text{XIDZ}\left(Ln\_sa,Ln\_ds\;,1\right). \end{array}$$
Formula (11) shows that the borrower influence coefficient is the ratio of the number of satisfied borrowers to the number of dissatisfied borrowers, and its initial value is 1.
  (12) $$\begin{array}{c} Ir\_flu=\text{IFTHENELSE}\left(\left.\text{In\_}avsh>Ln\_epa,-1\times \text{MIN}\left(XI\;DZ\left(\left(Ir\_ref-Ir\_dn\right),\times \left(\text{In\_}avsh-Ln\_epa\right)\text{,}2\times Ln\_epa\text{,0}\right)\text{,}Ir\_ref-Ir\_dn\right),\text{MIN}\left(XI\;DZ\left(Ir\_up-Ir\_ref\right)\times \left(\text{In\_}epa-\text{In\_}avsh\right),2\times \left(\text{In\_}avsh,0\right),Ir\_up-Ir\_ref\right)\right)\right). \end{array}$$
Formula (12) assumes that when the total investable amount is 3 times or more the total expected loan, then the platform interest rate reaches the lowest value. When the total investable amount equals the total expected loan, then the interest rate fluctuation coefficient is 0. When the total expected loan is 3 times or more the total investable amount, then the platform interest rate reaches the highest value. When the total investable amount is greater than the total expected loan, then the value of the interest rate fluctuation coefficient is negative. When the total investable amount is less than or equal to the total expected financing amount, then the value of the interest rate fluctuation coefficient is positive.
  (13) $$\begin{array}{c} Ir\text{\_in}=Ir\_ref+Ir\_flu. \end{array}$$
Formula (13) shows that the investment interest rate is the sum of the benchmark interest rate and interest rate fluctuation coefficient.
(2) The system dynamics model of flow rate variable formulas is as follows:
  (14) $$\begin{array}{c} \text{In\_}dsr=\text{In\_}ds\;k\times \text{In\_}epr. \end{array}$$
Formula (14) shows that the increase rate of dissatisfied investors is the product of the number of project expiration lenders and the dissatisfaction coefficient.
  (15) $$\begin{array}{c} \text{In\_}hdr=\frac{\text{MIN}\left(\text{In\_}avsh\text{,}Ln\text{\_}epa\right)}{\left(Ae\text{\_in}\right)}. \end{array}$$
Formula (15) shows that the increase rate of actual investors is the minimum value between the total investable amount and the expected loan amount, which is divided by the average investment amount.
  (16) $$\begin{array}{c} \text{In\_}rgr=\text{DELAY MATERIAL}\left(\text{MAX}\left(\text{In\_}lkr\times \text{In\_}rgk\times \text{In\_}fuk,0\right),\text{In\_}lkt,0,0\right). \end{array}$$
Formula (16) uses the DELAY MATERIAL to calculate the increase rate of investment registrants, which refers to the wait-and-see potential investors who registered after the wait-and-see period. The actual number of registrants is the product of the increase rate of wait-and-see investors, the registration coefficient, and the investor influence coefficient. The first 0 is given to avoid negative invalid results, the second 0 indicates that the initial value of the registrants' increase rate is 0, and the third 0 indicates that the function returns 0 in the absence of the first argument.
  (17) $$\begin{array}{c} \text{In\_}lkr=\text{In\_}lka\times \text{In\_}fuk. \end{array}$$
Formula (17) shows that the increase rate of wait-and-see investors is the product of the amount of investment general concern and the investor influence coefficient.
  (18) $$\begin{array}{c} \text{In\_}epr=\frac{Ln\_exr\times \left(Ln\_avm\right)}{\left(Ae\_\text{in}\right)}. \end{array}$$
Formula (18) shows that the increase rate of the number of investors whose projects are due is the product of the number of borrowers whose project are due and the average amount of borrowing, which is divided by the average amount of investment.
  (19) $$\begin{array}{c} \text{In\_}wtr=\text{INTEGER}\left(\text{MAX}\left(\left(\text{In\_}rg\times 0.02+\text{In\_}rgr\right)\times \text{In\_}wtk\times \text{In\_}fuk\times \left(\frac{Ir\text{\_in}-Ir\_dn}{Ir\text{\_up}-Ir\_dr}\right),0\right)\right). \end{array}$$
Formula (19) shows that the increase rate of effective investors is the product of the potential effective investors and the investment coefficient, the investor influence coefficient, and the interest rate influence coefficient. It is assumed that 2% of investors who have registered but not invested will be converted into potential investors and that all newly registered investors are potential investors. The interest rate influence coefficient is the difference between the real investment rate and the lowest interest rate, divided by the highest and lowest interest rates. The minimum value of this variable is 0, which indicates that the higher the investment interest rate is, the faster the increase rate of effective investors is.
  (20) $$\begin{array}{c} \text{In\_}sar=\text{In\_}epr\times \text{In\_}sak \end{array}$$
Formula (20) shows that the increase rate of satisfied investors is the product of the number of investors whose projects are due and investor satisfaction.
  (21) $$\begin{array}{c} Ln\_exr=\text{DELAY MATERIAL}\left(Ln\_rpr,Ln\_tm,0,0\right). \end{array}$$
Formula (21) shows that the increase rate of borrowers whose projects are due comes from the increase rate of the number of successful borrowers. After the term of loan, borrowers' projects are due.
  (22) $$\begin{array}{c} Ln\_wtr=\text{DELAY MATERIAL}\left(\text{INTEGER}\left(Ln\_a\;dk\;\times Ln\_apr\right),Ln\_a\;dt,\text{0},\text{0}\right). \end{array}$$
Formula (22) shows that the speed of loan approval is the product of the loan approval coefficient and the speed of the loan application. After the loan approval time, some loans are approved.
  (23) $$\begin{array}{c} Ln\_rpr=\text{INTEGER}\left(\text{MAX}\left(XI\;DZ\left(Ae\_\text{in}\times \text{In\_}hdr,Ln\_avm,\text{0}\right),\text{0}\right)\right). \end{array}$$
Formula (23) shows that the increase rate of successful borrowers is the product of the increase rate of the average investment amount and the number of actual investors, which is divided by the average loan amount.
  (24) $$\begin{array}{c} Ln\_rgr=\text{DELAY MATERIAL}\left(\text{INTEGER}\left(Ln\_lkr\times Ln\_rgk\times Ln\_ifk\right),Ln\_lkt,\text{0},\text{0}\right). \end{array}$$
Formula (24) shows that the increase rate of loan registrants is transformed from the product of the increase rate of loan followers, the registration coefficient of the borrower, and the influence coefficient of the borrower after a certain delay time, which is the loan wait-and-see time.
  (25) $$\begin{array}{c} Ln\_apr=\text{INTEGER}\left(\left(Ln\_rgr+Ln\_rg\times 0.001\right)\times Ln\_apk\times \left(\frac{Ir\_up+Pf\_rt-Ln\_ir}{Ir\_up-Ir\_dn}\right)\right). \end{array}$$
Formula (25) shows that the increase rate of loan applicants is obtained by the product of potential borrowers, the loan application coefficient, and the interest rate influence coefficient. The number of potential borrowers is 0.001 times the number of registered prospective borrowers, plus the rate of increase in borrowers registered. The influence coefficient of the interest rate consists of the difference between the highest interest rate plus the platform cost rate and the loan rate, which is divided by the difference between the highest interest rate and the lowest interest rate. The higher the loan rate is, the smaller the influence coefficient of the interest rate is, and the lower the number of loan applicants is.
  (26) $$\begin{array}{c} Ln\_lkr=\text{INTEGER}\left(Ln\_lka\times \left(1+Ln\_plk\right)\right). \end{array}$$
Formula (26) shows that the increase rate of loan followers is the product of the general number of loan followers and the sum of 1 plus the loan policy coefficient. A loan policy coefficient equal to 0 means no influence, a value greater than 0 means a positive influence, and a value less than 0 means a negative influence.
(3) The system dynamics model of level variable formulas is as follows:

(27) $$\begin{array}{c} \text{In\_}ds=\text{INTEG}\left(\text{In\_}dsr,0\right). \end{array}$$

Formula (27) shows that the number of dissatisfied investors is the integral of the increasing speed of dissatisfied investors, whose initial value is 0.

(28) $$\begin{array}{c} \text{In\_}rg=\text{INTEG}\left(\text{MAX}\left(\text{In\_}rgr-\text{In\_}wtr,0\right),0\right). \end{array}$$

Formula (28) shows that the number of investors who have registered but not invested is the integral of the difference between the growth rate of registrants and effective investors.

(29) $$\begin{array}{c} \text{In\_}ar=\text{INTEG}\left(\text{In\_}rgr,0\right). \end{array}$$

Formula (29) shows that the total number of investment registrants is the integral of the increase rate of investment registrants, and its initial value is 0.

(30) $$\begin{array}{c} \text{In\_}lk=\text{INTEG}\left(\text{MAX}\left(\text{In\_}lkr-\text{In\_}rgr,0\right),\text{In\_}lka\right). \end{array}$$

Formula (30) shows that the number of investment followers is the integral of the difference between the increase rate of investment followers and the increase rate of registrants, and its initial value is the number of general investment followers.

(31) $$\begin{array}{c} \text{In\_}ep=\text{INTEG}\left(\text{In\_}epr,0\right). \end{array}$$

Formula (31) shows that the total number of investors whose projects are due is the integral of the increase rate of investors whose projects are due, and its initial value is 0.

(32) $$\begin{array}{c} \text{In\_}hd=\text{INTEG}\left(\text{In\_}h\;dr-\text{In\_}epr,0\right). \end{array}$$

Formula (32) shows that the total number of holders of investment products is the integral of the difference between the increase rate of holders of investment products and the increase rate of investors whose projects due. Its initial value is 0.

(33) $$\begin{array}{c} \text{In\_}sa=INTEG\left(\text{In\_}sar,0\right). \end{array}$$

Formula (33) shows that the total number of satisfied investors is the integral of the increase rate of satisfied investors. Its initial value is 0.

(34) $$\begin{array}{c} \text{In\_}wt=INTEG\left(\left(\text{In\_}wtr+\text{In\_}sar-\text{In\_}hdr\right),0\right). \end{array}$$

Formula (34) shows that the total number of waiting investors is the integral of the difference of the sum between the increase rate of effective investors, the increase rate of satisfied investors, and the increase rate of actual investors. This actually means that all the satisfied investors are transformed into waiting investors and continue to wait for investment. Its initial value is 0.

(35) $$\begin{array}{c} \text{In\_}am=INTEG\left(\text{In\_}hd\text{r}\times Ae\text{\_in},0\right). \end{array}$$

Formula (35) shows that the total investment amount is the integral of the product of the increase rate of the number of actual investors and the average investment amount. Its initial value is 0.

(36) $$\begin{array}{c} Ln\_ds=INTEG\left(\text{INTEGER}\left(Ln\_exr\times Ln\_ds\;k\right),0\right). \end{array}$$

Formula (36) shows that the total number of unsatisfied borrowers is the integral of the product of the increased speed of borrowers whose loans have become due and the coefficient of unsatisfied borrowers. Its initial value is 0.

(37) $$\begin{array}{c} Ln\_rg=INTEG\left(\text{MAX}\left(Ln\_rgr-Ln\_apr,0\right),0\right). \end{array}$$

Formula (37) shows that the total number of borrowers who have registered and are waiting for a loan is the integral of the difference between the rate of increase of loan registrants and the rate of increase of loan applicants. Its initial value is 0.

(38) $$\begin{array}{c} Ln\_rp=INTEG\left(Ln\_rpr-Ln\_exr,0\right). \end{array}$$

Formula (38) shows that the number of borrowers who have repayments is the integral of the difference between the increase rate of borrowers who have repayments and the increase rate of borrowers whose loans have become due. Its initial value is 0.

(39) $$\begin{array}{c} Ln\_sa=INTEG\left(\text{INTEGER}\left(Ln\_exr\times Ln\_sak\right),0\right). \end{array}$$

Formula (39) shows that the total number of satisfied borrowers is the integral of the product of the increase rate of borrowers whose loans have become due and the coefficient of satisfied borrowers. Its initial value is 0.

(40) $$\begin{array}{c} Ln\_wt=INTEG\left(Ln\_wtr-Ln\_rpr,0\right). \end{array}$$

Formula (40) shows that the total number of borrowers waiting for funds is the integral of the difference between the increase rate of loan approval and the increase rate of borrowers who have obtained funds. Its initial value is 0.

(41) $$\begin{array}{c} Ln\_am=\text{INTEG}\left(Ln\_rpr\times Ln\_avm,0\right). \end{array}$$

Formula (41) shows that the total loan amount is the integral of the increase rate of the number of borrowers who obtain funds and the average loan amount. Its initial value is 0.

(42) $$\begin{array}{c} Ln\_ap=\text{INTEG}\left(Ln\_apr-Ln\_wtr,0\right). \end{array}$$

Formula (42) shows that the total number of loan applicants is the integral of the difference between the increase rate of the number of loan applicants and the increase rate of the number of borrowers whose loans are approved. Its initial value is 0.

(43) $$\begin{array}{c} Ln\_ar=\text{INTEG}\left(Ln\_rgr,0\right). \end{array}$$

Formula (43) shows that the total number of loan registrants is integral to the increase rate of the number of loan registrants. Its initial value is 0.

(44) $$\begin{array}{c} Ln\_lk=\text{INTEG}\left(Ln\_lkr-Ln\_rgr,0\right). \end{array}$$

Formula (44) shows that the total number of loan followers is the integral of the difference between the increase rate of the number of loan followers and the increase rate of the number of loan registrants. Its initial value is 0.

## 4. Algorithm Design and Implementation Steps

1. **Step 1:** Start the Vensim system.
2. **Step 2:** Draw the dynamic flow diagram of each subsystem.
3. **Step 3:** Establish the system dynamics model of the auxiliary variable formulas (5)–(13) and input it into the Vensim system.
4. **Step 4:** Establish the system dynamics model of the flow rate variable formulas (14)–(26) and input it into the Vensim system.
5. **Step 5:** Establish the system dynamics model of the level variable formulas (27)–(44) and input it into the Vensim system.
6. **Step 6:** Set the initial value of the start time variable, the end time variable, and the simulation step length, which are represented by “days.”
7. **Step 7:** Assign values to the auxiliary variables and enter them into the Vensim system.
8. **Step 8:** Run the Vensim system and start the simulation.
9. **Step 9:** Call the values of the auxiliary variables.
10. **Step 10:** Call the system dynamics model of the auxiliary variable formulas (5)–(13).
11. **Step 11:** Call the system dynamics model of the flow rate variable formulas (14)–(26).
12. **Step 12:** The Vensim system dynamically calculates the current values of the rate variables based on the value of the auxiliary variables and the model (14)–(26).
13. **Step 13:** Call the system dynamics model of the level variable formulas (27)–(44).
14. **Step 14:** Vensim dynamically calculates the current value of each level variable based on the values of the auxiliary variables and the model (27)–(44).
15. **Step 15:** Simulate a drawing of the online lending platform.
16. **Step 16:** If *t* < *T*, then *t*=*t*+*λ*; return to Step 8. Otherwise, the algorithm ends.

## 5. Validation of the System Dynamics Model

In the previous sections, we constructed the system dynamics model of an online lending platform. In this section, we use data from China's online lending industry to assign values to variables, simulate the operation of the online lending platform, and verify the rationality of the variable results, the data consistency, and the parameter sensitivity.

### 5.1. Parameter Variable Assignment

To observe the effect of long-term operation of the model, that is, to simulate the development of the online lending platform in 10 years, the initial time of model simulation is 1 (*t*=1), and the end time is 3650 (*T*=3650). Simulation step *λ*=1, which indicates the once-daily simulation calculation. We assign values to the parameter variables of models (5)–(44) with reference to the actual situation of China's online lending industry. The details are shown in Table 1.

After completing the variable assignment, we start running the simulation to obtain the values of other variables over time.

### 5.2. Logical Consistency Test

The correct simulation result data have the following relations:

1. Total investment amount = total loan amount.
2. Number of investors who hold investment products  ^*∗*^ average investment amount = number of borrowers who have loans  ^*∗*^ average loan amount.
3. According to the statistical results of the interest rate of online lending home, the investment interest rate shows a downward trend and finally fluctuates by approximately 10%. Since the parameter setting of the model is based on the data of the online lending home, the interest rate trend of the model should also be consistent with the data of the online lending home.

The simulation results of the model are shown in Figures 5–24.

The simulation results show that the interest rate trend of the model is consistent with the actual interest rate trend of China's lending industry. Thus, the relevant equation is correct, and the logical correctness of the model is explained.

### 5.3. Sensitivity Test

**Figure 10:**
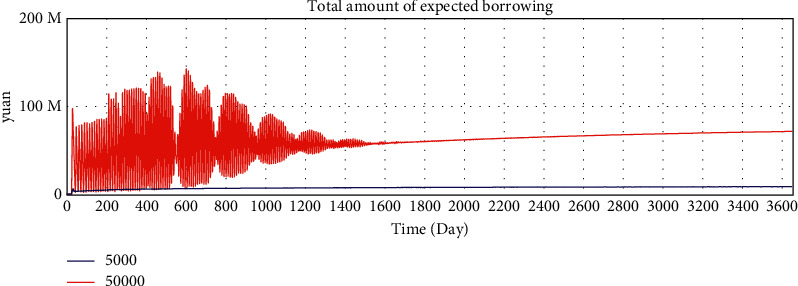
Sensitivity test—total amount of expected borrowing.

**Figure 11:**
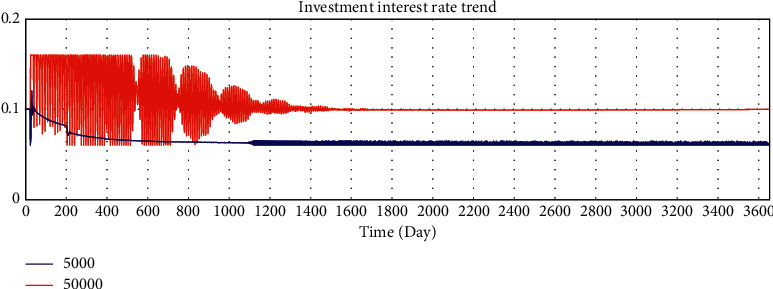
Sensitivity test—investment interest rate trend.

**Figure 12:**
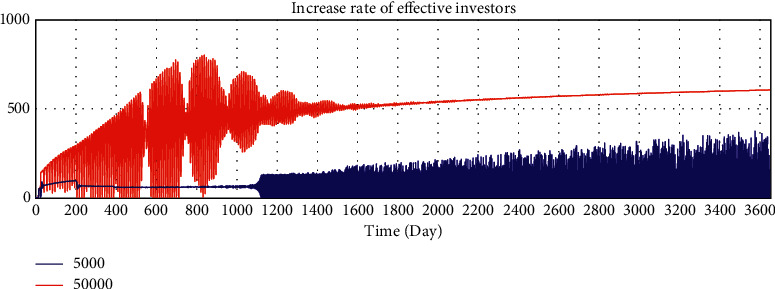
Sensitivity test—increase rate of effective investors.

**Figure 13:**
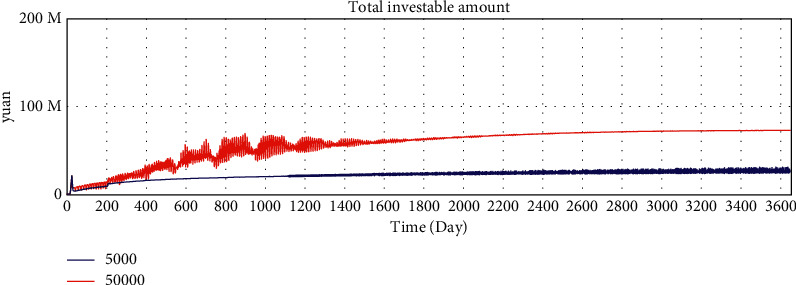
Sensitivity test—total investable amount.

**Figure 14:**
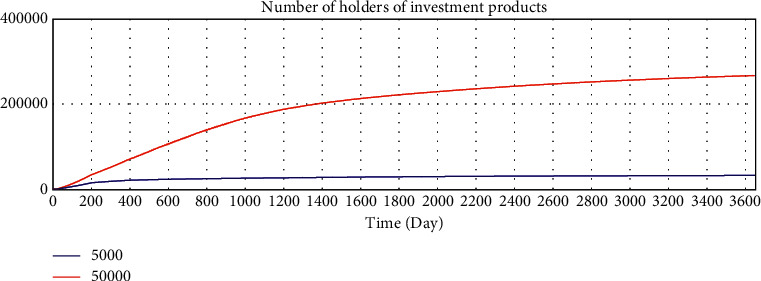
Sensitivity test—number of holders of investment products.

**Figure 15:**
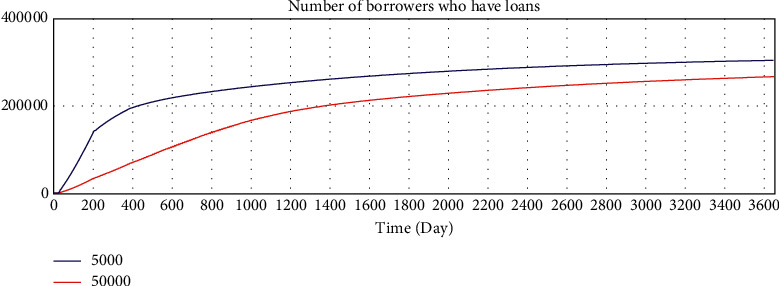
Sensitivity test—number of borrowers who have loans.

By adjusting the variable values of the model, we verify whether the running results of the model change in a corresponding manner, and we verify the sensitivity of the model to variables. The average loan amount is adjusted from 50000 yuan to 5000 yuan to test the sensitivity of the model. After adjustment, the simulation results are shown in Figures 10–15.

According to the analysis of the simulation results, when the average loan amount decreases, the expected total financing amount decreases, the investment interest rate decreases, the increase rate of loan applicants increases, the increase rate of effective investors decreases, the total investable amount decreases, the number of investment product holders decreases, and the number of borrowers who have loans increases. On the one hand, the sensitivity of the model to parameter changes is verified; on the other hand, the correctness of the logic function of the model is verified.

## 6. Data Analysis Based on Vensim Simulation Technology

### 6.1. Variable Analysis of Simulation Results

According to the online lending platform's publicly available data, we select the values of some variables, including the number of investors registered, the number of holders of investment products, the increase rate of investment registrants, the total amount of investment, the total number of borrowers registered, the total number of borrowers who have loans, and the investment interest rate. Then, we compare their values with the data of the real online lending platform and evaluate the reasonableness of the results.

**Figure 16:**
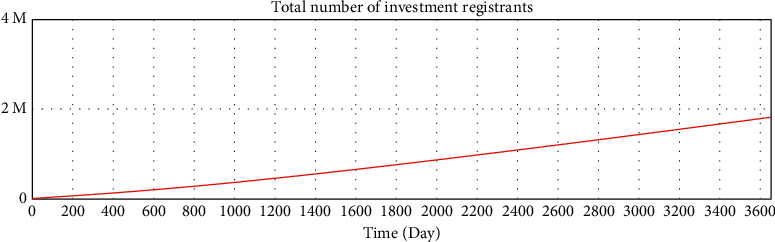
Test of variables—total number of investment registrants.

**Figure 17:**
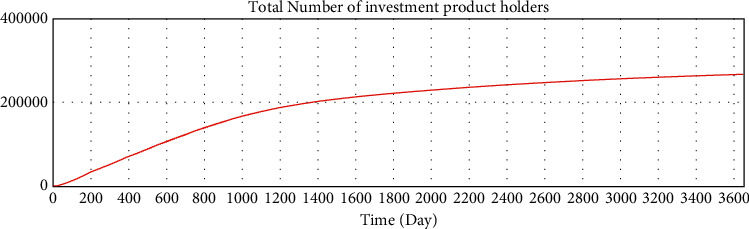
Test of variables—total number of investment product holders.

**Figure 18:**
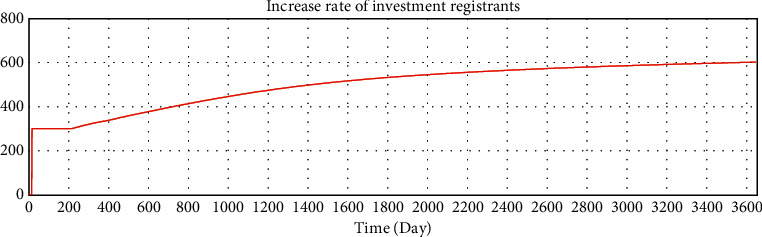
Test of variables—increase rate of investment registrants.

**Figure 19:**
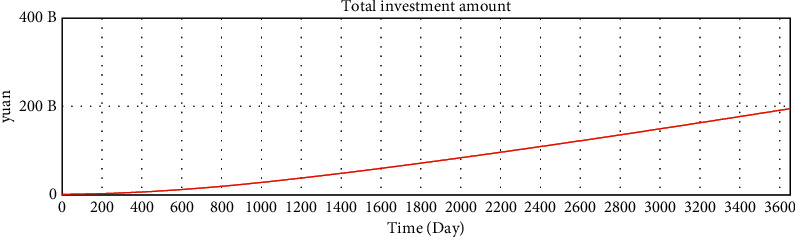
Test of variables—total investment amount.

**Figure 20:**
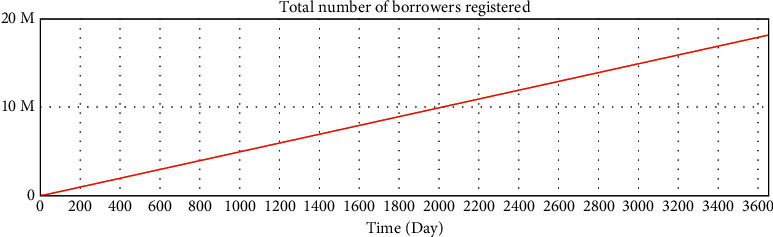
Variable test—total number of borrowers registered.

**Figure 21:**
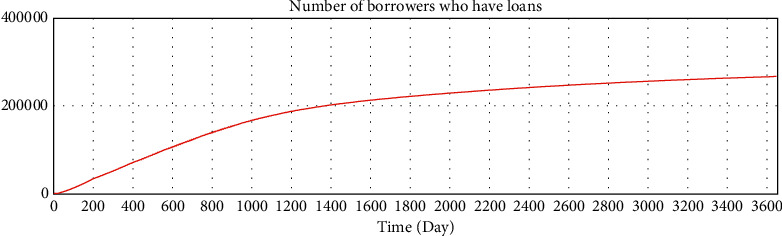
Test of variables—number of borrowers who have loans.

We use Vensim to simulate the data. After 10 years of development, the simulation data of the online lending platform in this model are as follows: the total number of registered investors is approximately 1.78 million, the number of holders of investment products is approximately 253,000, the total number of registered borrowers has reached 20 million, the number of holders of loan products is approximately 253,000, and the total transaction amount has reached 179 billion. The details are shown in Figures 16–21.

According to the data disclosed on the official website of A, platform A was launched in 2012. By the end of February 2019, the total transaction amount of the platform had reached 130.8 billion yuan, the total balance of outstanding loans had reached 14.5 billion yuan, the investors holding investments had numbered approximately 220,000, and the borrowers obtaining loans had numbered approximately 370,000.

According to the data disclosed on the official website of B, platform B was launched in March 2009. By the end of March 2019, there had been 2.74 million registered users, 133,000 borrowers who had obtained loans, and 486,000 investors who had held investment projects. The total outstanding balance of loans was 18.7 billion yuan, and the total transaction amount was 452.8 billion yuan.

Because the operation modes of different online lending platforms vary greatly and the development of online lending platforms is affected by policies, economic environment, management strategies, and other aspects, it is difficult to accurately quantify simulations. The simulation model is compared with the operation data of platform A and platform B, and the simulation results have certain practical significance in terms of the number of investors, the total outstanding balance of loans, the number of registrants, and soon.

### 6.2. Scenario Simulation of the Impact of Emergencies on Interest Rates

As soon as the policy changes, some investors will withdraw their money and stop investing. This paper simulates this scenario by modifying the variable equation of the investment coefficient and investors who are waiting for investing. The details are as follows:

Investment coefficient is as follows: In_*wtk*=IFTHENELSE( (Time > 2000):AND:(Time < 2060), 02,043).

This means that between 2000 and 2060, the percentage of registrants willing to invest falls from 0.43 to 0.2.

Investors who are waiting to invest are as follows: In_*wtk*=INTEG(IFTHENELSE((Time > 2000) : AND : (Time < 2060), In_*wtr* − In_hdr,In_*wtr*+In_*sar* − In_*hdr*), ，0).

This means that investors whose projects are due withdraw money and suspend investment.

In this paper, the investment coefficient is reduced from 0.43 to 0.3, 0.2, and 0.1 to simulate the impact of minor emergencies, general emergencies, and major emergencies on the online lending platform, respectively.

**Figure 22:**
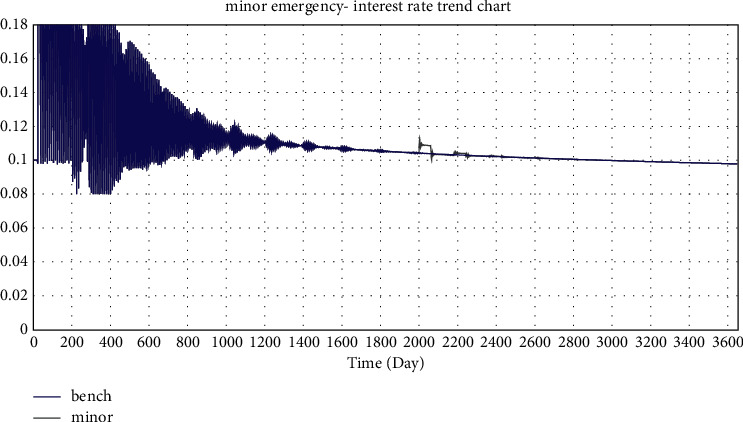
Simulation of the minor emergency—interest rate trend chart.

**Figure 23:**
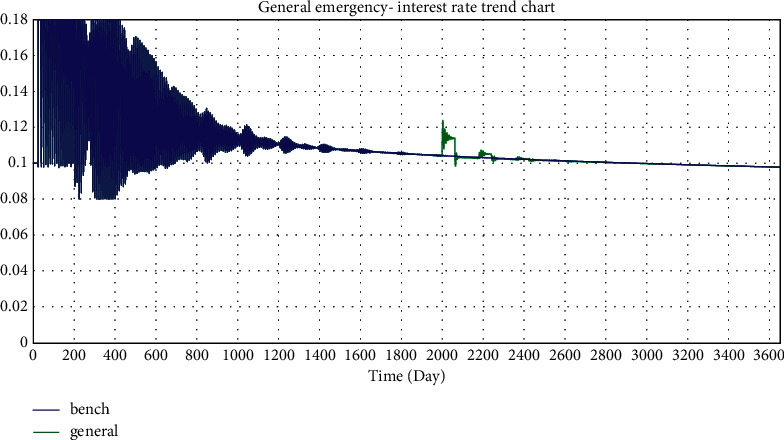
Simulation of the general emergency—interest rate trend chart.

**Figure 24:**
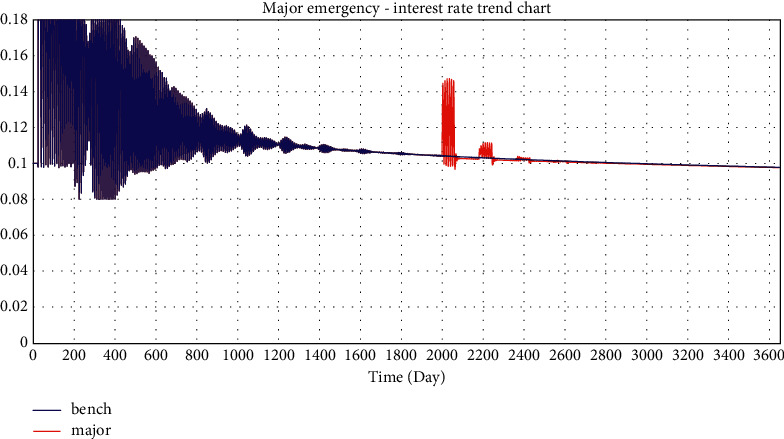
Simulation of major emergency—interest rate trend chart.

Selected simulation data and graphs of the impact of emergencies on investment interest rates are shown in Tables 2 and 3 and Figures 22–24.

According to the simulated data and graphs, when an emergency occurs, the platform interest rate will rise rapidly and fluctuate greatly; however, eventually, the interest rate will return to its normal state. When major emergencies occur, interest rates rise directly to the highest rate, with a wider impact range of 0.096–0.18. In addition, the emergency period is followed by three cyclical rate hikes, which decrease and eventually return to normal. It can be seen from the simulation data that there are three interest rate hikes with an interval of approximately 180 days, which is exactly the parameter value of the project duration set in the simulation. This is consistent with WDZJ's statistical chart on the interest rate trend of China's online lending industry. Although Chinese regulators issued several regulatory documents on the online lending industry between 2015 and 2019, the long-term interest rate trend of the industry was not significantly affected and ultimately remained at approximately 10%.

## 7. Conclusion

The current academic research on online lending platforms mainly focuses on industry characteristics, operation management, industry supervision, platform risks, and other aspects, while there are few studies that focus on the interest rate formation mechanism of online loan platforms. In the field of financial lending, the interest rate is directly related to asset quality and lending risk. Therefore, it is of great theoretical and practical significance to study the interest rate formation mechanism of online lending platforms. On the surface, the interest rate of an online lending platform is the subjective pricing of lending funds set by the platform. In fact, the interest rate is affected by the supply and the demand of funds and is closely related to the amount, term, and cost expectation of the funds on both the supply and demand sides. In essence, the interest rate is the result of the interaction of various factors during the operation of the online lending platform, which is affected by investors, borrowers, loan term, operating costs, regulatory policies, and other factors.

In this paper, we use system theory to analyze the structure and function of the online lending platform, extract the key factors of the platform operation, innovatively build the system dynamics model of the platform, and verify the model. Verification results show that the model can effectively reflect the changes in investors, borrowers, and interest rates of online lending platforms. We use this model to simulate the impact of emergencies on the interest rates of online lending platforms. The research results show that when an emergency occurs, the platform investment interest rate will rise rapidly and fluctuate widely; however, eventually, the interest rate will return to its normal state. This finding is consistent with the interest rate trend of China's online loan industry released by WDZJ, which further verifies the effectiveness of the model.

In short, this paper innovatively constructed a complete and effective system dynamics model of online lending platforms, but it also has some shortcomings. For example, this paper only uses the model to simulate the evolution trend of interest rate and does not analyze the rule of the number of investors and lenders. In the future, we will improve the model and extend the application scenarios of the model, such as loan term management, loan amount management, and interest rate management.

## Figures and Tables

**Figure 5:**
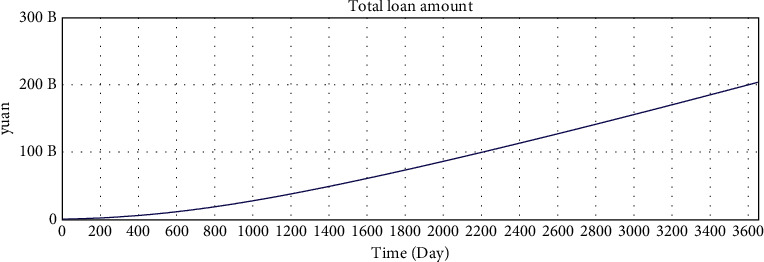
Consistency test—total loan amount.

**Figure 6:**
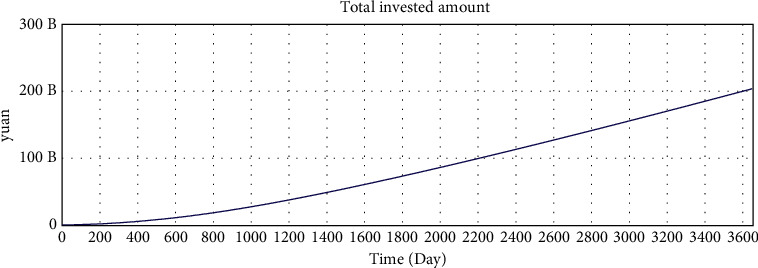
Consistency test—total invested amount.

**Figure 7:**
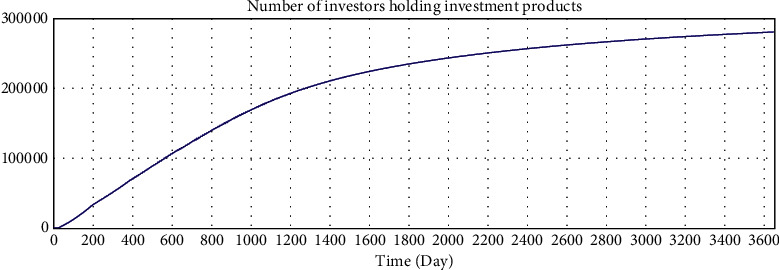
Consistency test—number of investors holding investment products.

**Figure 8:**
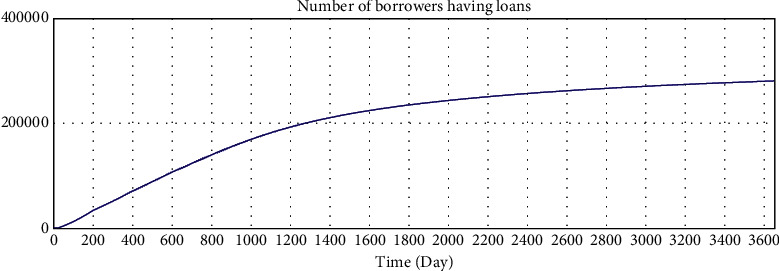
Consistency test—number of borrowers having loans.

**Figure 9:**
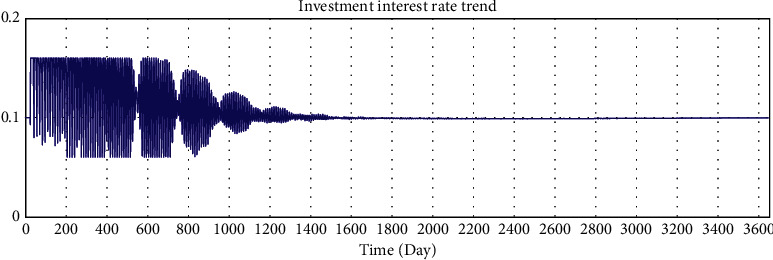
Consistency test—investment interest rate trend.

**Table 1:** Variables summary table.

SN	Variable name	Type
1	*In*_*lka*	Auxiliary variable
2	*In*_*pal*	Auxiliary variable
3	*In*_*lkt*	Auxiliary variable
4	*In*_*rgk*	Auxiliary variable
5	*In*_*wtk*	Auxiliary variable
6	*In*_*sak*	Auxiliary variable
7	*In*_*dsk*	Auxiliary variable
8	*Ae*_in	Auxiliary variable
9	*Ln*_*lka*	Auxiliary variable
10	*Ln*_*lkt*	Auxiliary variable
11	*Ln*_*rgk*	Auxiliary variable
12	*Ln*_*apk*	Auxiliary variable
13	*Ln*_*tm*	Auxiliary variable
14	*Ln*_*ir*	Auxiliary variable
15	*Ln*_*ifk*	Auxiliary variable
16	*Ln*_*avm*	Auxiliary variable
17	*Ln*_*epa*	Auxiliary variable
18	*Ln*_*adk*	Auxiliary variable
19	*Ln*_*adt*	Auxiliary variable
20	*Ln*_*sak*	Auxiliary variable
21	*Ln*_*dsk*	Auxiliary variable
22	*Ln*_*plk*	Auxiliary variable
23	*Ir*_*up*	Auxiliary variable
24	*Ir*_*dn*	Auxiliary variable
25	*Ir*_*ref*	Auxiliary variable
26	*Ir*_in	Auxiliary variable
27	*Ir*_*flu*	Auxiliary variable
28	*In*_*fuk*	Auxiliary variable
29	*Pf*_*rt*	Auxiliary variable
30	*In*_*asvh*	Auxiliary variable
31	*In*_*lkr*	Flow rate variable
32	*In*_*rgr*	Flow rate variable
33	*In*_*wtr*	Flow rate variable
34	*In*_*h* *dr*	Flow rate variable
35	*In*_*sar*	Flow rate variable
36	*In*_*ds* *r*	Flow rate variable
37	*In*_*eps*	Flow rate variable
38	*In*_*lkr*	Flow rate variable
39	*In*_*fuk*	Flow rate variable
40	*In*_*apr*	Flow rate variable
41	*In*_*wtr*	Flow rate variable
42	*Ln*_*rpr*	Flow rate variable
43	*Ln*_*exr*	Flow rate variable
44	*In*_*lk*	Level variable
45	*In*_*rg*	Level variable
46	*In*_*wt*	Level variable
47	*In*_*h* *d*	Level variable
48	*In*_*sa*	Level variable
49	*In*_*ds*	Level variable
50	*In*_*am*	Level variable
51	*Ln*_*lk*	Level variable
52	*In*_*ar*	Level variable
53	*Ln*_*rg*	Level variable
54	*In*_*rp*	Level variable
55	*In*_*ep*	Level variable
56	*In*_*ap*	Level variable
57	*Ln*_*sa*	Level variable
58	*Ln*_*ds*	Level variable
59	*Ln*_*ar*	Level variable
60	*Ln*_*am*	Level variable
61	*Ln*_*wt*	Level variable

**Table 2:** Parameter assignment and description.

SN	Parameter name	Variable name	Variable value	Description
1	Average loan amount	*Ln*_*avm*	50000 yuan	The average loan amount of all borrowers on the platform is 50000 yuan
2	Average investment amount	*In*_*am*	50000 yuan	The average investment amount of all investors on the platform is 50000 yuan
3	Benchmark interest rate	*In*_*ref*	10%	The platform sets the recommended interest rate
4	Platform cost rate	*Pf*_*rt*	3%	The platform sets the cost rate
5	Minimum interest rate	*Ir*_*dn*	7%	The platform sets the minimum investment interest rate
6	Highest interest rate	*Ir*_*up*	18%	The platform sets the highest investment interest rate
7	Coefficient of investment	*In*_*wtk*	0.5	The value ranges from 0 to 1, indicating the proportion of the investment registrants participating in the actual investment
8	Investment policy regulation coefficient	*In*_*pla*	0	The value range is [−1, 1]. A value less than 0 indicates that the policy has a negative impact on investors, which leads to a decrease in investors; a value greater than 0 indicates that the policy has a positive impact on investors, which leads to an increase in the number of investors. A value of 0 indicates no impact on investors
9	Investor registration coefficient	*In*_*rgk*	0.03	The value ranges from 0 to 1, which indicates the proportion of investment followers who become registrants
10	Satisfied borrower coefficient	*Ln*_*sak*	0.5	The value range [0-1] represents the proportion of borrowers who are satisfied with the service of the platform
11	Online lending borrowing policy coefficient	*Ln*_*plk*	0	The value range is [−1, 1]. A value less than 0 means that the policy has a negative impact on borrowers, which leads to a decrease in the number of borrowers. A value greater than 0 means that the policy has a positive impact on borrowers, which leads to an increase in the number of borrowers. A value of 0 means there is no impact on the borrowers
12	Number of general followers	*In*_*lka*	10000 persons	10,000 people pay attention to the online lending platform every day (visit information related to P2P sites is available at chinaz.com)
13	Loan approval time	*Ln*_*adt*	3 days	The time interval between an application for a loan and the approval of the loan
14	Loan review factor	*Ln*_*adk*=30%	0.3	The value ranges from 0 to 1, which indicates the proportion of loan applicants who are qualified for loans
15	Loan application coefficient	*Ln*_*apk*	0.6	The value ranges from 0 to 1, which indicates the proportion of loan registrants applying for loans
16	Borrower registration coefficient	*Ln*_*rgk*	0.5	The value range is [0-1], which indicates the proportion of loan followers who register on the platform
17	Loan wait-and-see time	*Ln*_*lkt*	20 days	The time interval from the beginning of the loan wait-and-see period to registration
18	Investor satisfaction factor	*In*_*sak*	0.6	The value range is [0-1], which indicates the proportion of investors who are satisfied with the platform

**Table 3:** Emergency simulation—investment rate simulation data.

Time (year)	Investment rate: small emergency scenarios	Investment rate: general emergency scenarios	Investment rate: major emergency scenarios	Investment interest rate: reference benchmark
1999	0.102207	0.102207	0.102207	0.102207
2000	0.113438	0.13074	0.137085	0.102302
2001	0.128309	0.174591	0.18	0.102229
2002	0.133802	0.18	0.18	0.10206
2003	0.137062	0.18	0.18	0.101843
2004	0.13155	0.18	0.18	0.101636
2005	0.123803	0.151351	0.18	0.101554
2006	0.116208	0.127829	0.18	0.10161
2007	0.110205	0.103079	0.18	0.101794
2008	0.110326	0.095844	0.18	0.10202
2009	0.11484	0.132468	0.18	0.102167
2010	0.121157	0.154913	0.157082	0.102182
2011	0.126291	0.176678	0.133459	0.102054
2012	0.128271	0.18	0.109219	0.101875
2013	0.127211	0.172388	0.0962937	0.101707
2014	0.123828	0.154704	0.158322	0.101596
2015	0.119754	0.129936	0.18	0.101595
2016	0.116625	0.10561	0.18	0.101671
2017	0.115634	0.0998262	0.18	0.101834
2018	0.117079	0.129777	0.18	0.101981
2019	0.119883	0.152967	0.18	0.102056
2020	0.122627	0.173256	0.18	0.102036
2021	0.124209	0.178974	0.168392	0.101912
2022	0.124254	0.170342	0.141543	0.101764
2023	0.122958	0.153888	0.117719	0.101651
2024	0.121039	0.130235	0.0960768	0.101596
2025	0.119309	0.109093	0.14408	0.101611
2026	0.118479	0.104562	0.177372	0.101698
2027	0.118768	0.129454	0.18	0.101819
2028	0.119933	0.152788	0.18	0.101943

## Data Availability

The data in this article are all from the Online Loan Home (https://top.chinaz.com/site_www.wdzj.com.html). The Online Loan Home is a global open Internet platform, so the data are publicly available at the observatory.
